# Evolutionary trajectory of SARS-CoV-2 genome shifts during widespread vaccination and emergence of Omicron variant

**DOI:** 10.1038/s44298-023-00007-z

**Published:** 2023-11-14

**Authors:** Kaitlyn Gayvert, Sheldon McKay, Wei Keat Lim, Alina Baum, Christos Kyratsous, Richard Copin, Gurinder S. Atwal

**Affiliations:** Regeneron Pharmaceuticals Inc., Tarrytown, NY 10091 USA

**Keywords:** High-throughput screening, Pathogenesis

## Abstract

Understanding the adaptation of SARS-CoV-2 is critical for the development of effective treatments against this exceptionally successful human pathogen. To predict the emergence of new variants that may escape host immunity or increase virulence, it is important to characterize the biological forces driving its evolution. We conducted a comprehensive population genetic study of over thirteen million SARS-CoV-2 genome sequences, collected over a timeframe of ~3 years, to investigate these forces. Our analysis revealed that during the first year of the pandemic (2020 to 2021), the SARS-CoV-2 genome was subject to strong conservation, with only 3.6% of sites under diversifying pressure in the receptor binding domain (RBD) of the Spike protein. However, we observed a sharp increase in the diversification of the RBD during 2021 (8.1% of sites under diversifying pressure up to 2022), indicating selective pressures that promote the accumulation of mutations. This period coincided with broad viral infection and adoption of vaccination worldwide, and we observed the acquisition of mutations that later defined the Omicron lineages in independent SARS-CoV-2 strains, suggesting that diversifying selection at these sites could have led to their fixation in Omicron lineages by convergent evolution. Since the emergence of Omicron, we observed a further decrease in the conservation of structural genes, including M, N, and the spike proteins (13.1% of RBD sites under diversifying pressure up to 2023), and identified new sites defining future potential emerging strains. Our results exhibit that ongoing rapid antigenic evolution continues to produce new high-frequency functional variants. Sites under selection are critical for virus fitness, and currently known T cell epitope sequences are highly conserved. Altogether, our study provides a comprehensive dynamic map of sites under selection and conservation across the entirety of the SARS-CoV-2 genome.

## Introduction

The severe acute respiratory syndrome coronavirus 2 (SARS-CoV-2), the etiologic agent of the coronavirus disease 2019 (COVID-19) global pandemic, has prompted an extensive effort to understand its adaptation mechanisms. Scientists and medical professionals worldwide have sequenced the SARS-CoV-2 genome from patient isolates, and their findings have been rapidly disseminated through curated data repositories such as the global initiative on sharing all influenza data (GISAID, https://www.gisaid.org)^1,2^. This unprecedented level of data sharing has provided a unique dataset critical to determine transmission patterns and identify variants that may be associated with virulence and disease severity.

The biological forces driving the conservation and diversity of SARS-COV-2 genome are currently not well understood. In the past, understanding these forces has been critical to inform treatment development against other life-threatening infections^3–7^. While mutations emerge randomly in the genome, the accumulation and loss of mutations can be driven by changes within the host environment. For example, vaccination or emergence of a new variant can lead to sequence diversity or conservation of circulating viral genomes. During infection, pressure to escape recognition by antigen-targeting immune factors such as antibodies and T lymphocytes can lead to diversifying selection of viral antigens, while emergence of a dominant lineage in the viral population can reduce the diversity of the viral quasispecies and increase genome conservation.

Characterizing which regions in the genome are conserved or diverse with an emphasis on antigenic targets has critical implications for immune surveillance, drug development and resistance. To date, both B cell and T cell immune responses have been reported to play critical roles in controlling SARS-CoV-2 infections^8^. B cells produce both anti-SARS-CoV-2 neutralizing and non-neutralizing antibodies, which comprise the polyclonal responses. Many of the most potently neutralizing anti-SARS-CoV-2 antibodies target the receptor-binding domain (RBD) of the viral spike protein, inhibiting the binding to the ACE2 cell host receptor^9–19^. Antigen-specific T cells recognize short peptide epitopes generated by proteolysis of pathogen proteins, bound to HLA molecules on the surface of antigen-presenting cells. SARS-CoV-2 reactive memory CD4^+^^20^ and CD8^+^^21^ T cells have also been reported in unexposed individuals, suggesting that pre-existing cross-reactive T cells could drive disease outcomes in infected patients.

In this study, we leveraged 13,128,166 SARS-CoV-2 genome sequences collected worldwide from the time of the first reported sequence in late 2019 to October 2022 to assess and predict the evolution of the virus and the future of the pandemic. First, we highlighted amino acid mutations spreading in the community at high frequency. We then established the evolutionary relationship of SARS-CoV-2 isolates and employed population genetic analyses to determine the selective forces driving genetic diversity in SARS-CoV-2 at both protein and amino acid levels. To explore the impact of broad infection, vaccination and emergence of Omicron lineages on SARS-CoV-2 evolution, we generated a quarterly time series dataset that revealed how evolution of the SARS-CoV-2 genome has changed across three defining time periods of the pandemic, namely, the pre-vaccine era (2020), the post-vaccine era (2021) and the emergence of omicron lineages (2022). Finally, we present a comprehensive genetic analysis of the currently known SARS-CoV-2 B and T cell epitopes to determine whether all SARS-CoV-2 antigens represent potential target of immune escape.

## Results

### Comprehensive analysis of sequence variation in SARS-CoV-2

**Fig. 1:**
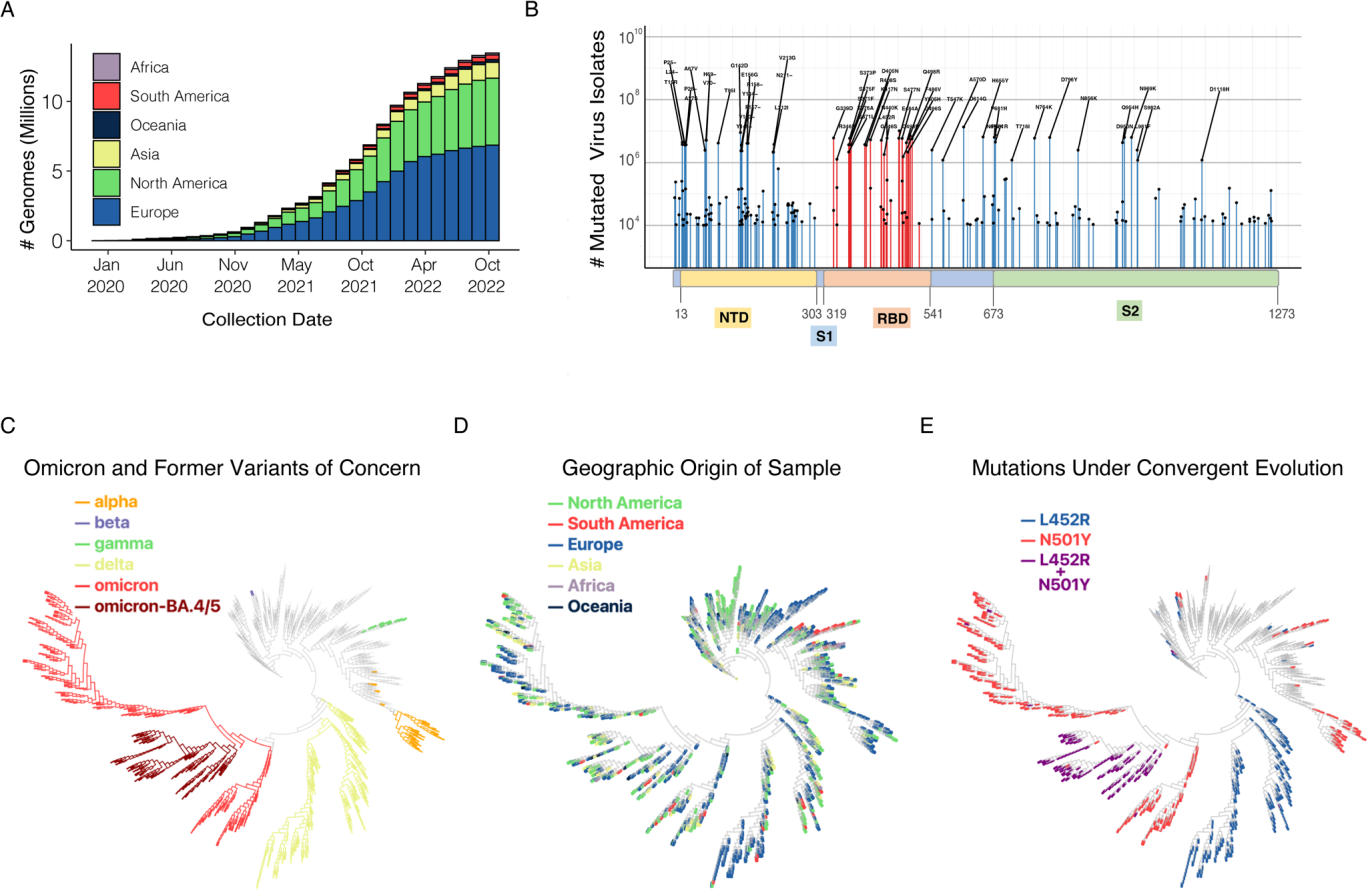
SARS-CoV-2 diversity across geographic regions and phylogenetic lineages. **A** Cumulative count of SARS-CoV-2 genome sequences in GISAID by collection date and continent of sample origin as of November 1, 2022. **B** Distribution of high frequency mutations (HFM) in the spike protein sequence. The RBD and NTD domains are highlighted in orange and yellow, respectively. Mutations in the RBD are indicated in red. Amino acid change of mutations identified in at least a million GSAID sequences are shown. For residues with high deviation from the wild type sequence (eg. 417, 452, 484, 501, 614), the most common amino acid change is indicated. **C** SARS-CoV-2 maximum-likelihood phylogeny of high-diversity 1000-genome subset. The tree is outgroup rooted with the related bat CoV genome RATG13. The root branch length is truncated to emphasize relationships amongst SARS-CoV-2 genomes. Distribution of haplotypes of the six previous and current variants of concern (Alpha, Beta, Gamma, Delta, Omicron, Omicron BA.4/5), mapped onto the phylogeny. **D** The geographic origin of each of the sequences is denoted by branch color. **E** Distribution of mutations under convergent evolution (L452R, N501Y), mapped onto the phylogeny.

Since the first SARS-CoV-2 genome sequence was reported in early January 2020, there have been over thirteen million sequences deposited to GISAID (https://www.gisaid.org/)^1,2^. Each genome sequence is associated with comprehensive patient-related metadata that can be used to determine the time of infection and the geographical origin of the virus isolates (Fig. 1A). We compared the identity of all coding sequences retrieved from 13,128,166 curated genomes. We found that 26% of all mutations were identified in only one given isolate (singleton). However, there were 1154 high frequency mutations (HFM) that were shared across at least 10,000 isolates occurring at 10% of total sites genome-wide (1016 out of a total 9726 sites) (Supplementary Table S1). A disproportionate number of HFM were found in ORF7a (39%; 47 out of a total 122 sites within the gene), ORF8 (31%; 38 / 122 sites), and ORF3a (29%; 79 / 276 sites).

The spike protein exhibited enrichment of sequence diversity (14%; 175/1274 sites) compared to the entire genome sequence. HFM identified within the spike protein were found to be accumulating within the N-terminal domain (NTD) (24%; 71/291 sites) (Fig. 1B), while the RBD had a similar number of HFM (13%; 28/223 sites) when compared to the full spike protein and more HFM compared to the genome as a whole.

### SARS-CoV-2 diversity across phylogenetic lineages

Detailed phylogenetic analysis of a data set on the order of millions of genome samples is computationally challenging due to the combinatorial complexity of evaluating relationships between all samples. To obtain a computationally feasible data set, we developed a down-sampling method to select a subset of 10,000 representative genomes (see Methods)^22,23^. Genome-based phylogenetic analysis resolved relationships among samples and the evolution of the virus in relation to geographic distribution. Many lineages contained SARS-CoV-2 isolates from several continents, indicating limited or no apparent restrictions between lineages and local populations (Fig. 1C, D). Comparison of the diversity of individual genomes by lineage allowed identification of mutations following a pattern of convergent evolution (Fig. 1E). These mutations included N501Y and L452R, which were identified independently in genomes from at least 6 and 7 major lineages, respectively (Table 1). This suggests emergence of both mutations occurred as a result of similar selective forces in independent individuals rather than linear transmission. Since phylogeny-defining mutations can be critical to understand virus biology, we further examined diversifying and purifying selection and functional studies of how mutations may impact SARS-CoV-2 biology.

**Table 1:** RBD sites under convergent evolution.

Effect	HFM	Associated major variants	Increased ACE2 binding affinity	Decreased ACE2 binding affinity	Reduced neutralization by convalescent sera	Reduced neutralization by therapeutic antibodies
Increased binding affinity and reduced neutralization	L452*	delta, epsilon, iota, kappa, lambda, delta, omicron	**X**		**X**	
Increased binding affinity	N501Y	alpha, beta, gamma, theta, mu, omicron	**X**			
	T478K	delta, omicron	**X**			
Reduced neutralization	E484*	beta, gamma, zeta, eta, kappa, iota, theta, mu, omicron			**X**	**X**
	K417*	beta, gamma, delta, omicron		**X**	**X**	**X**
	R346*	mu, omicron				**X**

(*) refers to multiple amino acid changes at this site.

### Selective landscape of the SARS-CoV-2 genome

Bayesian estimates of the SARS-CoV-2 genome mutation rate are in the order of 10^−3^–10^−4^ nucleotide substitutions per site per year^24–26^, with reported fluctuations over time^26,27^. This rate is low compared to other human coronaviruses and RNA viruses^25^, suggesting that purifying selection substantially shapes the SARS-CoV-2 genome. To characterize the selective forces driving SARS-CoV-2 gene sequence diversity, we identified sites evolving under natural selection by applying a Bayesian codon model, FUBAR^23^. Codon substitution models are used to identify sites that are evolving significantly faster (diversifying selection) or slower (purifying selection) than expected. Quickly spreading mutations under positive selection, especially those that arise independently across multiple lineages via convergent evolution, can be identified through these approaches by their elevated nonsynonymous (dN) versus synonymous (dS) substitution rates. Similarly, sites where the reference alleles are conserved and mutations fall exclusively on shallow branches of the phylogeny are typically under purifying selection, as evidenced by low dN/dS ratios (≪1), and are more likely to negatively impact the fitness of the virus^28^.

**Fig. 2:**
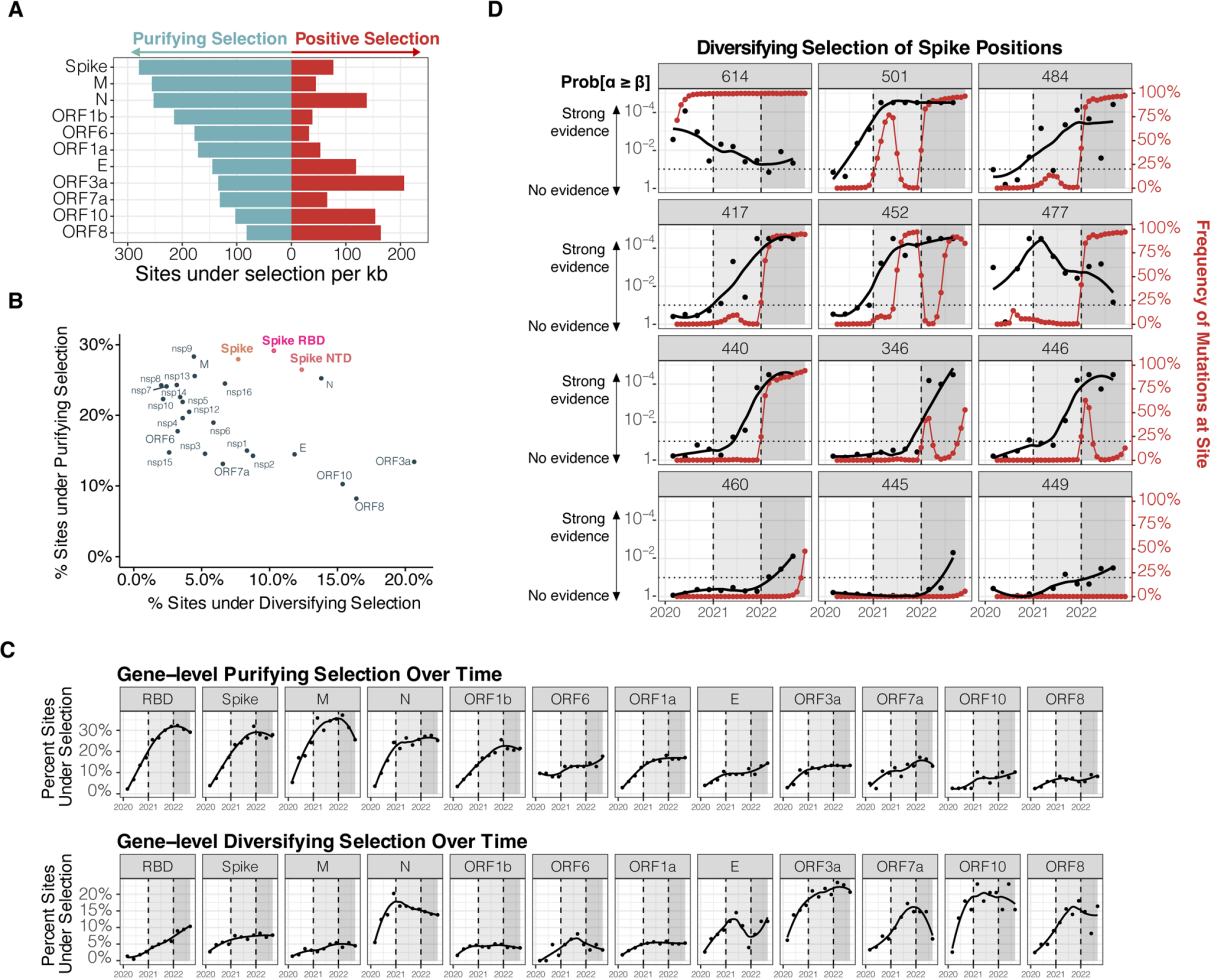
Amino acid sites under natural selection in SARS-CoV-2 genome. **A** Gene length normalized counts (counts per kb) of sites under significant purifying selection (light blue bars) and diversifying selection (red bars). **B** Scatterplot of percent of sites under purifying selection vs percent of sites under diversifying selection in each protein. While overall the genome was under strong purifying selection, a substantial number of sites were identified as under diversifying selection in certain regions (eg. ORF3a, ORF10, N). **C** Percentage of sites under purifying (top panel) and diversifying (bottom panel) selection per gene across the three defining time periods of the pandemic: (1) the pre-vaccine era, (2) the post-vaccine era, and (3) the emergence of omicron lineages. **D** Level of evidence (1—probability of selection) (black lines and points) and global frequency of mutations (red lines and points) at selected sites under diversifying selection in spike across the three defining time periods of the pandemic: (1) the pre-vaccine era, (2) the post-vaccine era, and (3) the emergence of omicron lineages. alpha: synonymous mutation rate; beta: non-synonymous mutation rate; Prob[alpha ≥ beta]: 1—probability of diversifying selection at the site.

Overall, a gene-based measure of selection confirmed strong purifying selection acting on the SARS-CoV-2 genome but also identified a substantial number of sites under diversifying selection in several genes (Fig. 2A). Amongst structural and accessory protein-encoding genes, *N, E, ORF3a, ORF8, and ORF10* genes showed an elevated number of sites under diversifying selection, indicating that these genes were under distinct selective forces when compared to the rest of the genome. Early stop codons are amongst the most frequent HFM in ORF3a (Q57H) and ORF8 (Q27*)^29^ and ORF10 is no longer treated as a protein-coding gene^30^. Interestingly, most of these genes have been implicated in the suppression of the innate immune response, particularly in the suppression of type one interferon signaling (ORF8), blocking autophagy (ORF3a^31^), and downregulation of antigen presentation (ORF8^32^).

When compared to other proteins within the SARS-CoV-2 genome, the spike protein and its RBD region were under some of the highest levels of purifying selection observed, along with the M, Nsp7-ORF1a, and Nsp9-ORF1a proteins (Fig. 2B and Supplementary Fig. S1A). While similar levels of HFM were present in the Spike protein and RBD, we observed that the RBD was under elevated diversifying selection (10%; 23/223 sites), including the seven of the most frequently mutated sites (339, 417, 452, 477, 478, 484, 501) (Supplementary Fig. S1B, C), which may be consistent with the pressure imposed by the immune system on this domain. This indicated that despite having a globally conserved genome, evolutionary analysis at the single amino acid level revealed sites with significant diversity. A comprehensive list of all sites under selection is provided as a Supplementary Table S2.

### Gene level selection over time

To better understand how levels of selection on SARS-CoV-2 genome have changed over time, we repeated our evolutionary analysis to generate a quarterly time series dataset. These timepoints span across three temporal windows, representative of three milestones of the recent pandemic, i.e.,: (1) the pre-vaccine era (2020), (2) the post-vaccine era (2021), and (3) the emergence of Omicron lineages (2022) (see Methods). At the gene level, we observed a sharp shift in the selective forces acting on SARS-CoV-2 proteins. While initially, many genes underwent an intensification of purifying selection over time (most notable for the Spike, M, N, and ORF1b), this trend disappeared and often reverted with the widespread utilization of vaccination worldwide and emergence of Omicron lineages, respectively (Fig. 2C and Supplementary Fig. S1D). Conversely, diversification of the Spike-RBD continued to increase across the three time periods (Table 2), indicating intensification of the selective pressures promoting accumulation of mutations within this given region.

**Table 2:** RBD sites under diversifying selection over time.

Spike Site	Pre-Vaccine	Post-Vaccine	Post-Omicron
339	Neutral	Neutral	Diversifying
346	Neutral	Neutral	Diversifying
367	Diversifying	Diversifying	Diversifying
371	Purifying	Neutral	Diversifying
376	Neutral	Neutral	Diversifying
384	Neutral	Diversifying	Diversifying
405	Neutral	Neutral	Diversifying
408	Neutral	Neutral	Diversifying
414	Neutral	Diversifying	Diversifying
417	Neutral	Diversifying	Diversifying
439	Diversifying	Diversifying	Neutral
440	Neutral	Diversifying	Diversifying
445	Neutral	Purifying	Diversifying
446	Diversifying	Diversifying	Diversifying
449	Neutral	Diversifying	Diversifying
450	Neutral	Neutral	Diversifying
452	Neutral	Diversifying	Diversifying
460	Neutral	Neutral	Diversifying
477	Diversifying	Diversifying	Diversifying
478	Neutral	Diversifying	Diversifying
484	Diversifying	Diversifying	Diversifying
486	Neutral	Neutral	Diversifying
490	Neutral	Diversifying	Diversifying
493	Neutral	Diversifying	Diversifying
494	Diversifying	Diversifying	Diversifying
496	Neutral	Neutral	Diversifying
498	Neutral	Diversifying	Diversifying
501	Diversifying	Diversifying	Diversifying
505	Neutral	Neutral	Diversifying
522	Diversifying	Diversifying	Diversifying

Notably, we observed that the diversifying selection acting on sites of mutations that later would define Omicron lineages (417, 440, 446, 452, 501, and 484) increased at the onset of the post-vaccine era. These sites remained under strong diversifying selection even in the cases where the mutations disappeared from circulation (417, 452, and 501) (Fig. 2D). This led to the transient fixation of these mutations in independent SARS-CoV-2 lineages and ultimately to their fixation by convergent evolution in Omicron lineages. Since the emergence of Omicron, new sites under diversifying selection have appeared (445, 449, and 460), indicating that the evolution of the virus is still ongoing and should be vigilantly monitored (Fig. 2D and Supplementary Fig. S2). A comprehensive table detailing how selective pressures at each site genome-wide have changed over time is provided as Supplementary Table S3.

### Impact of selective forces on SARS-CoV-2 infectivity and antibody neutralization

**Fig. 3:**
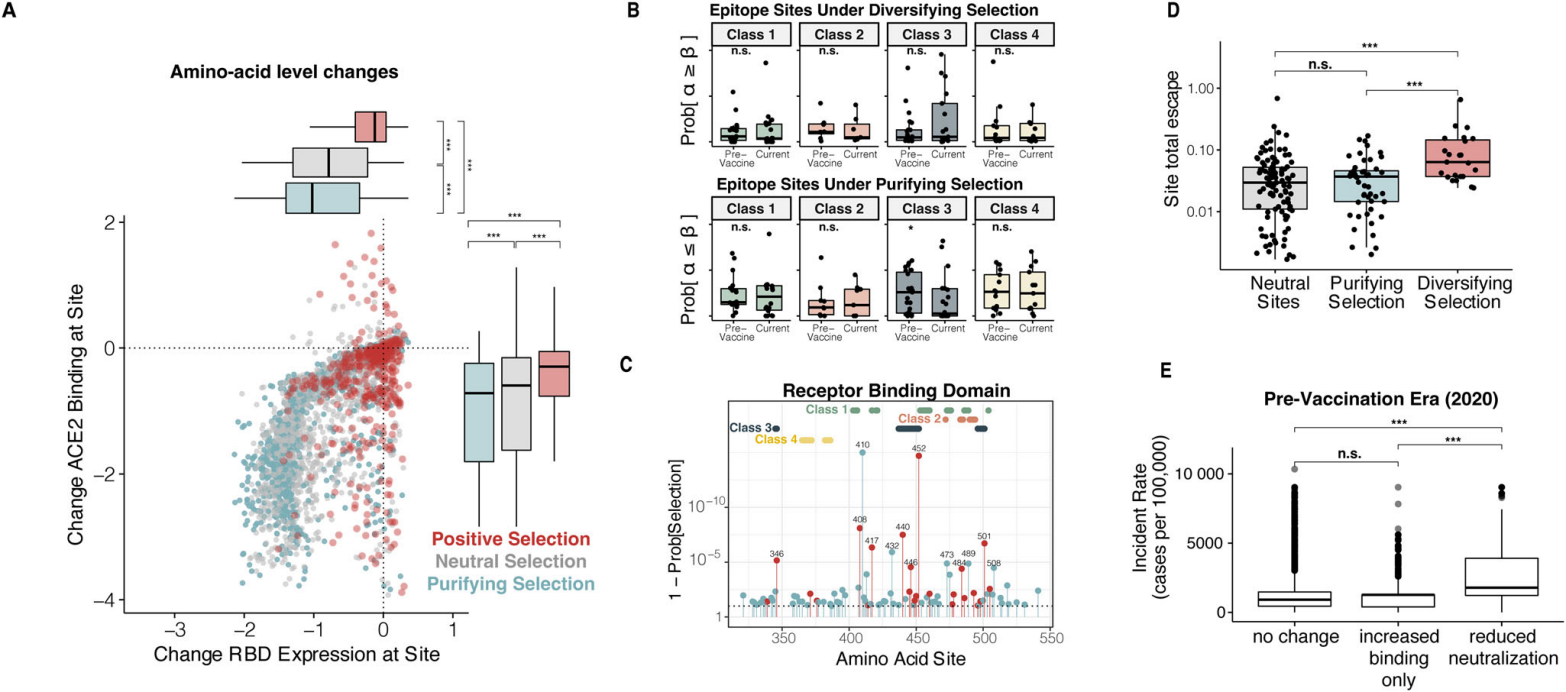
Impact of selective forces on SARS-CoV-2 infectivity and antibody neutralization. **A** For each possible amino acid substitution within the RBD, the effect of the mutation on RBD expression (x-axis) and ACE2 binding (y-axis). Each substitution is colored according to whether its associated site is under diversifying (red), purifying (blue), or neutral (gray) selection. **B** Levels of diversifying and purifying selection of each antibody epitope class site during the pre-vaccine and current post-Omicron eras. **C** Sites detected to be under purifying selection (blue) or diversifying selection (red) within the RBD of the S protein. Overlayed are the sites associated with class 1 (green), 2 (orange), 3 (navy blue), and 4 (yellow) antibody epitopes. **D** Deep mutational scan site total escape from convalescent plasma values for sites under diversifying (red), purifying (blue), or neutral (gray) selection. **E** Boxplot of the incident rates (cases per 100,000) for each sample in the pre-vaccination era (collected in 2020), grouped by whether that sample contained a mutation that reduced neutralization (G446V or mutations at sites L452, E484, or K417), increased ACE2 binding without any impact on neutralization (T478K or mutations at sites N501 or S477), or had no effect on neutralization and binding (A520S or mutations at site A522).

To further characterize how sites under selection within the RBD may relate to the pressures of the host environment, we first determined the impact of mutations under selection on infectivity. We assessed the functional constraints of ACE2 binding using previously published deep mutational screens that measured the impact of mutations on affinity for ACE2 binding and protein folding stability, as estimated by RBD expression^33^. Substitutions at sites under purifying selection (88.4%) had the strongest negative impact on ACE2 binding affinity and protein stability (Fig. 3A and Supplementary Fig. S3A). Conversely, sites under diversifying selection had less effect on binding and stability (61.1% of sites) (Fig. 3A). Since this screen was limited at estimating increased binding affinity due to global epistasis estimation, N501Y was the only HFM in the RBD that demonstrated a substantial increase ACE2 binding affinity in this analysis. However, additional HFM have been reported to enhance binding affinity (N440K^34^, L452R^35,36^, S477N^37^, S477I^38^, T478K^39^, and S494P^40^), and these changes have been linked to increased infectivity and transmissibility^35–37,41^.

We next looked to assess how sites under selection impacted antibody neutralization. We compared how the site-level estimates of selection related to the four major classes (binding regions) of SARS-CoV-2 neutralizing antibodies. Each class varies in its ability to block ACE2 (class 1, 2) and its binding configuration to the RBD (class 1, 4: “up”-only; class 2, 3: “up” and “down”)^42^. While purifying selection applied to epitopes from all antibody classes, it had the strongest impact on epitopes from class 1 and class 4 antibodies (*p* = 0.0169) (Fig. 3B) and the lowest impact on epitopes from class 3 antibodies. Overall, we observed that diversifying selection acting on class 3 antibody epitopes increased during the post-vaccine era (Fig. 3B). However, epitopes from each of these classes contained at least one site under strong diversifying selection which have been shown to impact therapeutic antibodies (Fig. 3C). Indeed, class 1 (e.g., casirivimab, etesevimab), class 2 (e.g., bamlanivimab) and class 3 antibodies (e.g., C110) have been reported to be negatively impacted by mutations at the 417^43–46^, 484^43–47^, and 452^43,48–50^ sites respectively. The sites under diversifying selection significantly decreased antibody neutralization when compared to both sites under purifying (*p* = 2.5 × 10^−4^) and neutral selection (*p* = 6.2 × 10^−5^) (Fig. 3D and Supplementary Fig. S3B)^43,49–54^.

Finally, we analyzed the impact of RBD HFM at sites under diversifying selection and found that many of these mutations have an impact on ACE2 binding affinity (e.g., N501Y^55^), antibody neutralization (e.g., E484K/Q), or both (L452R/Q^35,36,49^). Interestingly, we found that many of these mutations have arisen at the same sites (R346, K417, L452, T478, E484, and N501) independently across multiple lineages (Table 1)^56^, indicating a common selective pressure acting on independent isolates. We also found that mutations at site 417 (K417N/Beta+Omicron; K417T/Gamma) and 501 were physically linked in 77% of isolates (5,658,304/7,338,018). Because mutations at site 417 reduced antibody neutralization at a cost of ACE2 binding, and N501Y increased ACE2 affinity, the data suggested that some mutations can combine to alleviate the detrimental effect of individual mutations.

Each of the currently characterized major variants contain at least one of the RBD HFM that are associated with increased infectivity. Additionally, all except Alpha also include a mutation that reduces antibody neutralization. The impact of the K417N/T and E484K on convalescent sera may have enabled the Beta and Gamma variants to spread in South Africa^57^ and Brazil^58^ despite high levels of seroprevalence in the late 2020 to early 2021 periods. To further investigate this, we annotated each isolate collected in 2020 with the incident rate (cases per 100,000) estimated from the geographic region and month from which the sample was collected, which we found to be a strong correlate with seroprevalence in the pre-vaccine era (Supplementary Fig. S4). We observed that the mutations that reduced antibody neutralization were found in areas with significantly higher seroprevalence (Fig. 3E). The Beta and Gamma, and later Omicron, variants have led to the loss of efficacy of both therapeutic antibodies (eg. bamlanivimab, etesevimab)^59^ and specific vaccines (e.g. ChAdOx1)^60^. Altogether, this suggests that the mutations associated with reduced neutralization have serious implications for public health.

### Selective pressures within candidate variable T cell epitopes

**Fig. 4:**
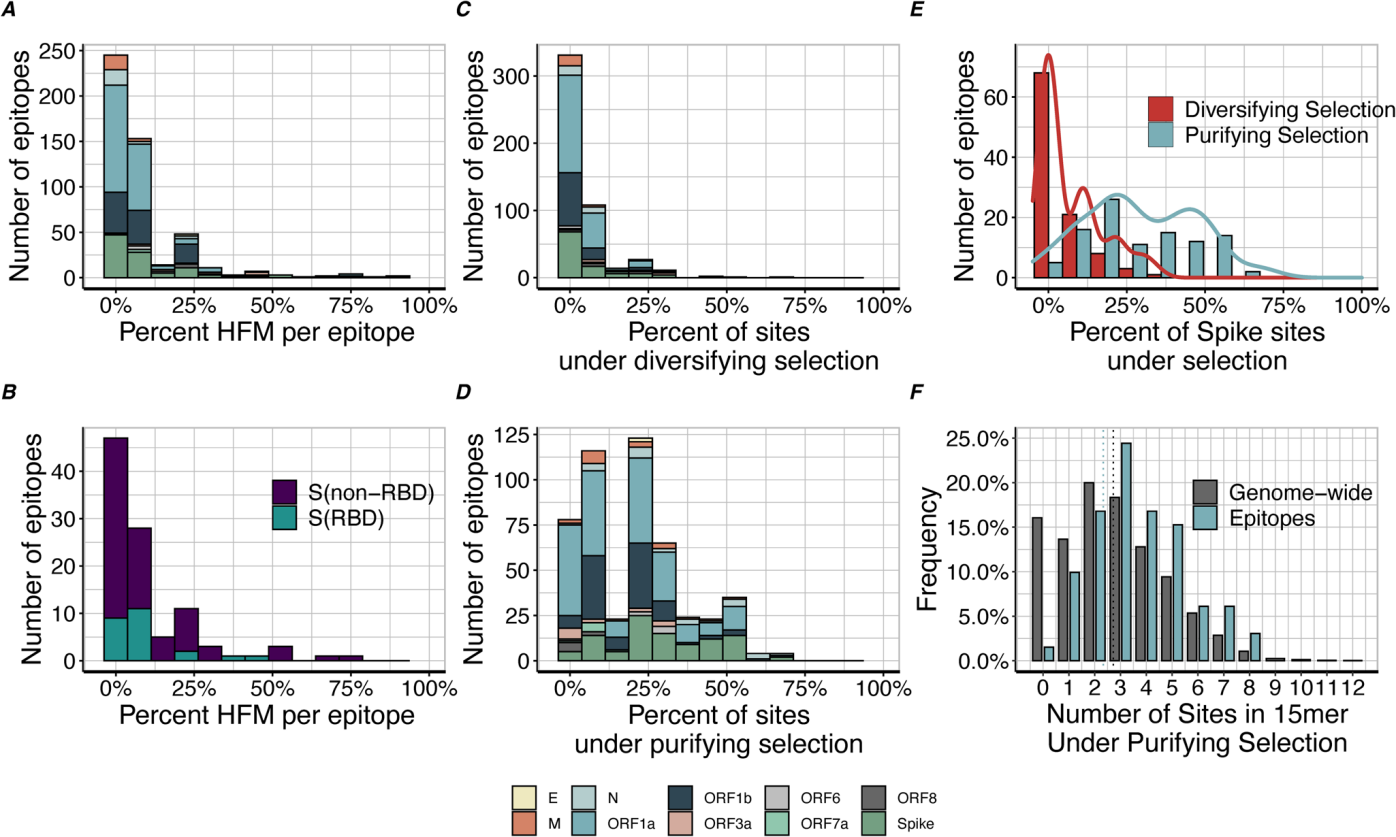
Selective pressures within candidate variable T cell epitopes. Distribution of the number of sequence high frequency mutations (found in 10,000+ isolates) observed in (**A**) putative memory CD8 + T cell epitope sequences from recovered COVID patients, and (**B**) epitopes from spike RBD and non-RBD regions. Number of putative memory CD8 + T cell epitope sequences from recovered COVID patients that contain sites under (**C**) diversifying and (**D**) purifying selection per gene. **E** Distribution of the number of sites under selection in epitopes, broken down by diversifying and negative selection in spike peptides. **F** Distribution of the number of sites under purifying selection in peptides (blue) and genome-wide (gray) 15-mers.

In order to determine if SARS-CoV-2 mutations occur in regions targeted by the human immune system, we examined the distribution of amino acid mutations across 142 previously reported putative memory CD4^+^ T cell epitopes from SARS-CoV-2-naïve individuals^20^ and 637 previously reported candidate memory CD8^+^ T cell epitopes from both SARS-CoV-2-naïve individuals and recovered COVID patients^21^. For each peptide, we determined all amino acid mutations observed in SARS-CoV-2 genomes present in GISAID. While all peptides had each amino acid position mutated in at least one SARS-CoV-2 isolate, the vast majority of epitopes had very little overlap with HFM (Fig. 4A, B and Supplementary Fig. S5A, B). Interestingly, the exception to this was ORF3a, which overlapped with HFM in 92% (12 / 13) of peptides.

To determine whether these putative T cell epitope sequences in SARS-CoV-2 strains were under selective pressure, we compared the number of sites under selection in the peptide sequences to background genome-wide levels of selection. Overall, we found that T cell epitope sequences were significantly enriched for sites under elevated purifying selection (*p* = 0.01), with a single epitope sequence on average containing 3% (*n* = 0–1) of their sites under diversifying selection and 20% (*n* = 2–3) of their sites under purifying selection (Fig. 4C–F). Notably, there were no significant differences in patterns of selection between either CD4 and CD8 epitopes nor between SARS-CoV-2-naïve and recovered COVID patients (Supplementary Fig. S5C–G). In addition to the absence of diversifying mutations, the spike, *ORF1a*, and *N* epitope sequences were significantly enriched for sites under purifying selection compared to the full genome (spike: *p* = 1.3 × 10^−12^, ORF1b: *p* = 0.04, N: *p* = 0.005) (Supplementary Fig. S6). Overall, the data indicates that T cell epitope sequences are under strong purifying selective pressures.

## Discussion

The Omicron variant is the most successful SARS-CoV-2 lineage to date as demonstrated by its rapid spread and dominance across the world, starting at the end of 2021. Its success was explained by the accumulation of RBD mutations in the binding footprint of the virus’s main entry host receptor (ACE2). Presence of such mutations within the RBD are known to potentially provide an evolutionary advantage by strengthening the virus infectivity, or by avoiding detection by neutralizing antibodies. One year following Omicron emergence, its origin remains unclear. Phylogenetic analysis has not identified any intermediary sequences between Omicron and its closest relatives and it did not reveal any straightforward evolutionary exit (frameshift or mutational profile) that could suggest that it descends from the Alpha, Beta, Delta or Gamma variants. However, defining mutations in Omicron lineages have been identified independently in strains early on during the pandemic. In our study, we showed that the majority of these mutations targeted sites that quickly became under diversifying selection months before the emergence of Omicron (sites: 417, 440, 446, 452, 501, and 484). More specifically, we found that diversifying forces acting on these sites intensified during the early utilization of vaccination worldwide, consistent with other reports^61,62^. Importantly, this demonstrates that sites identified under selective forces represent early clues to predict the genome makeup of the next wave of emerging SARS-COV-2 strains. With that in mind, our analysis showed that in addition to an accumulation of RBD sites remaining under strong diversifying selection, new RBD sites have also recently been targeted by newly arising diversifying selection (including 445, 449, and 460), indicating that the virus evolution is not slowing down and suggesting that it will lead to new SARS-CoV-2 variants.

The SARS-CoV-2 RBD is both the region mediating viral entry into the cell and the main target of most potent neutralizing anti-spike antibodies^63,64^. We showed that RBD sites under strong levels of diversification were often associated with increased ACE2 binding and infectivity, as well as reduced antibody neutralization consistent with selective pressure imposed by the immune system on this domain. We also observed that fixation of such mutations come at a fitness cost for the virus which can be compensated for by compensatory neutral mutations. For instance, while K417N/T mutations in the RBD increased immune escape^43,52^, it occurred at the fitness cost of reduced ACE2 binding affinity. Interestingly, we found that mutations at this site have not been successful in spreading without the addition of the compensatory mutation N501Y. Experimental studies have confirmed that while K417N alone results in a significant drop in ACE2 binding affinity, its combination with N501Y resulted in 3-fold stronger binding than wild-type (although still 2-fold less compared to N501Y alone)^65^.

Neutralizing monoclonal antibodies have also been isolated that target non-RBD epitopes^16,66^. For example, antibodies 4A8^66^ and 1–68^16^ have been reported to neutralize SARS-CoV-2 by targeting the NTD^67^, which was characterized by elevated levels of diversifying selection. Genomic polymorphisms, including the 144 deletion found in the Alpha variant, the 242–244 deletions in the Beta variant, and multiple NTD mutations found in the Delta variant, have demonstrated reduced sensitivity to anti-NTD neutralizing antibodies^68^.

Another significant finding of the present study is the identification of a substantial number of sites under diversifying and purifying selection outside of the spike protein. We found elevated and intensified levels of diversification in the *N, ORF3a, ORF7a, ORF8, and ORF10* genes. This can be explained by the roles these genes have been described to play in interacting with the immune system to block the innate immune response. Additionally, the N R203M mutation initially associated with the Delta variant was described to significantly increase mRNA production and delivery in to host cells and thus explain the increased transmissibility of this successful variant^69^. In our analysis, R203M mutation site was identified as being under selection, demonstrating that identification of sites under selection and subsequent phenotypic characterization of mutations occurring at these sites can help better understand the keys of SARS-CoV-2 success. Altogether, this suggests that the pressures outside of the spike are also important for fitness and warrant closer examination.

We also examined experimentally verified peptide epitopes derived from unexposed donors^20,21^ and recovered COVID patients^21^ for sequence mutations in SARS-CoV-2 and found that the vast majority were under strong conservation and did not accumulate sites under diversifying selection. In uninfected donors, SARS-CoV-2-reactive T cells can exhibit different patterns of immunodominance and frequently target *ORF1a* coding sequence, *nsp7*^8^. In the present study, we showed that *nsp7* is hyper-conserved and has very few sites actively under diversifying selection. This observation is coherent with the high level of identity between orthologous sequences of *nsp7* across multiple coronaviruses^70^ and helps explain why *nsp7*-specific T cells are detected and often dominant in unexposed donors as they may derive from exposure to other coronaviruses. Overall, data show that CD8^+^ and CD4^+^ T cell epitopes are still unaffected in Omicron genomes, suggesting that Omicron does not appear to escape T cell responses.

To understand the future burden of COVID-19, it is imperative to carefully monitor the mechanisms generating highly antigenically divergent variants and the circumstances underlying their emergence. New dominating variants impose a relentless burden on our immune system which in turn imposes pressure on the virus to select the next variant, which we predict will be as antigenically different from previous variants as possible to overcome host immunity. Our study informs patterns of antigenic evolution in infected and vaccinated individuals and gives us the tools to more reliably identify the currently forming variants that may yet emerge.

## Methods

### Sequence analysis, filtering, and alignment

There were approximately 13 million complete genome sequences deposited in GISAID^1,2^ as of November 12, 2022, which were downloaded along with their mutation calls and accompanying metadata. In order to reduce the number of genomes to a computationally tractable size, a two-stage down-sampling algorithm was developed to reduce the number of sequences to 2 million (Supplementary Table S4A). The first step in this process was collecting 100 randomly sampled accessions for each for each pango lineage. In cases where there less than 100 sequences for a lineage, all available sequences were used. The balance of the 2 million sequences were composed of randomly sampled sequences, irrespective of lineage, with sampling dates normalized by using 1000 sampled accessions for each month, where available, between December 2019 and October 2022. After the date-based sampling, the resulting subset was topped up to 2 million by randomly selecting the remaining accessions with no lineage or date constraints. The 2 million sequences were then filtered to meet the following quality control (QC) criteria: complete accession metadata (no missing location or sample collection date); less than 5% ambiguous DNA (N) nucleotide or translated amino (X) sequences; no runs of 6 or more consecutive Ns in the DNA or Xs in the protein sequence; the entire set of the canonical gene-based open reading frames (ORFs) for the coding genome with full length BLAST hits when searched against the reference genome; no ORFs truncated by premature stop codons. The premature stop codon constraint was relaxed for ORF8, which has a stop codon at amino acid position 27 in several lineages^29,30^. 820,056 sequences met all QC filters, which were used as the population sampled for downstream analysis steps. Intergenic regions were excluded.

For selection analysis, more computationally feasible subsets of ~10,000 genomes were further down-sampled (Supplementary Table S4B). The rapid spread of emerging variants of concern resulted in the majority of sampled genomes being from a dominant VOC lineage at any point in time. The sampling date distribution was simulated for the 10,000 sequence data sets by fitting the randomly sampled, QC-passed sequence count for each month of the pandemic to a log curve, favoring more recently samples genomes. Prior to this step, at least one randomly sampled representative of each lineage was preserved, with the balance of sequences composed of the date log curve-fitted random subsample. Using this method, up to 10,000 sequence sets were selected for timepoints. In some cases, sequences for a particular date range numbered less than 10, 000 and all were retained. For time points with greater than 10,000 QC-passed sequences available, the data were down-sampled to approximately 10,000 sequences. The following timepoints were used: 2020-03-31, 2020-06-30, 2020-09-30, 2020-12-31, 2021-03-31, 2021-06-30, 2021-09-30, 2021-12-31, 2022-03-31, 2022-06-30, 2022-09-30 (Supplementary Table S4B). Each timepoint represents the end of a 3-month quarter. Accessions sampled prior beginning of the first quarter were included in the 2020-03-30 subset. Available samples collected later than 2022-09-30 but available in the 2022-11-12 GISAID download were included in the 2022-09-30 subset. Six replicate down-sampled 10,000 sequence sets (Supplementary Table S4B) were generated for the final timepoint to evaluate reproducibility of selection analysis of the sequences sets resulting from our down-sampling methodology. For each of the timepoints, coding sequences of each full-length gene-based open reading frame were aligned with the codon-aware multiple sequence aligner virulign^71^. The aligned ORF sequences were then assembled into a full-length coding genomes.

### Phylogenetics

To guide the selection analysis (described below) for each of the timepoint data subsets, a whole-genome phylogenetic tree was inferred using fasttree^72^ with arguments -noml -nome –fastest, which was selected in order to optimize the tradeoff between accuracy and speed. To create the phylogeny annotated in Fig. 1, a smaller subset of 2000 QC-passed sequences (Supplementary Table S4B) was generated with unconstrained random sampling, except for preservation of at least one sequence of each lineage in the data subset. A more rigorous maximum-likelihood phylogenetic tree was inferred using fasttree^72^ for the 2000 genomes set. The tree was outgroup rooted using a GISAID accession EPI_ISL_402125 from December 2019. The maximum-likelihood tree was annotated with geographic, variant and mutation metadata using the ETE3 Python toolkit^71^. Images of annotated phylogenetic trees were rendered using the Python API to iTOL, the interactive Tree of Life^73^.

### Natural selection analysis

The selection test was performed using Fast Unconstrained Bayesian AppRoximation (FUBAR) method^23^ from the hypothesis testing using phylogenies (HyPhy) Suite (https://stevenweaver.github.io/hyphy-site). The analysis was conducted for each single gene. The prepared alignment and corresponding whole-genome phylogenies described above were inputted into FUBAR to infer nonsynonymous (dN) and synonymous substitution (dS) rates at a per-site basis and test whether dN was significantly different from dS. Probabilities that sites were under purifying or diversifying selection were reported separately, and sites with probability values >0.9 were considered to be under non-neutral (either purifying or diversifying) selection. This was repeated at the historical time points to understand the changes over time. For the purposes of interpretation, the historical quarters were binned into pre-vaccine (2020-03-31, 2020-06-30, 2020-09-30, 2020-12-31), post-vaccine (2021-03-31, 2021-06-30, 2021-09-30, 2021-12-31) and post-Omicron (2022-03-31, 2022-06-30, 2022-09-30) periods.

To assess whether the genome subsets were representative of the larger data set and that any results would be robust and reproducible, this analysis was repeated across six different replicates of the above-described down-sampling procedure. The consistency of the results was assessed using Pearson correlation of the log(1—probabilities) of diversifying and purifying selection across the full SARS-CoV-2 genome. There was a high degree of concordance among replicates (Supplementary Fig. S7), suggesting that these down-sampled subsets were sufficiently representative of the larger dataset and could be used to further investigate the evolution of the SARS-CoV-2 genome.

### Analysis of deep mutation scan data

Data from deep mutational scans were downloaded from the following sources:

*single_mut_effects.csv* file from https://github.com/jbloomlab/SARS-CoV-2-RBD_DMS (RBD binding, expression constraints), *final_variant_scores.csv* from https://github.com/jbloomlab/SARS-CoV-2-RBD_DMS_Omicron (RBD binding, expression constraints), and the *escape_data.csv* file from https://github.com/jbloomlab/SARS2_RBD_Ab_escape_maps (escape maps). The escape value was taken to be the average of “site_total_escape” across all convalescent plasma samples.

### Seroprevalence analysis

CDC seroprevalence estimates were downloaded from https://covid.cdc.gov/covid-data-tracker/#serology-surveillance. The John Hopkins daily incident rate estimates were downloaded from https://github.com/CSSEGISandData/COVID-19. For each US state, the relationship between seroprevalence estimates and incident rates was evaluated using Pearson’s correlation coefficient. Since these were highly correlated (*R* = 0.8), the incident rate was used as a proxy for seroprevalence. For each sample in GISAID, the incident rate at the month (Collection Date) and highest-resolution place (county when available, otherwise state/country) was annotated. The incident rates were used to compare trends where different sets of mutations were spreading. This analysis was restricted to the pre-vaccination era of sequences, as defined by the samples collected between June 2020 (when incident rates became reliable) and December 31, 2020 (when vaccinations began to ramp up in the United States).

### Analysis of CD4+ and CD8 + T cell epitope sequences

The list of 142 previously published memory CD4^+^ T cell epitope sequences and genome coordinates was downloaded from Supplementary Table 1 of ref. ^20^. The list of 637 previously published CD8^+^ T cell epitope sequences were downloaded from Supplementary Tables 4 and 7 of ref. ^21^ and each peptide sequence was aligned against the reference sequence using BLAST (accession: MN908947.3) to determine genome coordinates.

For each gene, we counted the total numbers of unique sites that were covered by epitope sequences and under diversifying/purifying selection. Significance was assessed using a fisher’s exact test comparing the number of sites under selection within sites targeted by candidate epitopes to genome-wide levels of selection. The number of sites under diversifying/purifying selection were computed for all 15-mers genome-wide and compared to epitope sequences using the one-sided Wilcoxon rank-sum test for each gene. The number of sites under diversifying/purifying selection were also computed for all 15-mers genome-wide and compared to epitope sequences using the one-sided Wilcoxon rank-sum test for each gene.

### Reporting summary

Further information on research design is available in the Nature Research Reporting Summary linked to this article.

## Supplementary information

Supplementary Materials

Reporting Summary

Supplementary Tables

Supplementary Data

## Data Availability

Sequences downloaded from the GISAID EpiCoV database are not available due to data-sharing restrictions, however, accession IDs for the sequences used in the analyses are provided in Supplementary Table S4. Data from deep mutational scans was downloaded from https://github.com/jbloomlab/SARS-CoV-2-RBD_DMS_Omicron (RBD binding, expression constraints) and https://github.com/jbloomlab/SARS2_RBD_Ab_escape_maps (escape maps). CDC seroprevalence estimates were downloaded from https://covid.cdc.gov/covid-data-tracker/#serology-surveillance. The John Hopkins daily incident rate estimates were downloaded from https://github.com/CSSEGISandData/COVID-19. Previously published T cell epitope sequences were downloaded from Supplementary Table 1 of ref. ^20^ and Supplementary Tables 4 and 7 of ref. ^21^.

## Code availability

Code will be made available at: https://github.com/regeneron-mpds/SARS-CoV-2_evolution.
