# Supplementary material for: Evolutionary trajectory of SARS-CoV-2 genome shifts during widespread vaccination and emergence of Omicron variant
Source: Npj Viruses. 2023 Nov 14;1:5. doi: 10.1038/s44298-023-00007-z (PMC11721106; doi:10.1038/s44298-023-00007-z)

Supplementary Materials for

Evolutionary trajectory of SARS-CoV-2 genome shifts during widespread vaccination and emergence of Omicron variant.

Kaitlyn Gayvert, Sheldon McKay, Wei Keat Lim, Alina Baum, Christos Kyratsous, Richard Copin*, Gurinder S Atwal*

*Corresponding authors. Email: richard.copin@regeneron.com and mickey.atwal@regeneron.com

This PDF file includes:

Supplementary Figure Legends
Supplementary Table Legends
Supplementary Figures S1-S7

SUPPLEMENTARY FIGURE LEGENDS

SUPPLEMENTARY FIGURE S1. Related to Figure 2.

(A) Protein length normalized counts (counts per kb) of sites under significant purifying selection (light blue bars) and diversifying selection (red bars) for the protein products of ORF1ab gene.

(B) Sites detected to be under purifying selection (left panel) or diversifying selection (right panel) in spike protein. Gray areas indicate the NTD and RBD regions.

(C) Scatterplot of the probability of diversifying selection at a site versus the number of isolates at that site with mutations.

(D) Percentage of sites under purifying (top panel) and diversifying (bottom panel) selection per ORF1ab protein product across the three defining time periods of the pandemic: (1) the pre-vaccine era, (2) the post-vaccine era, and (3) the emergence of omicron lineages.

SUPPLEMENTARY FIGURE S2. Related to Figure 2.

Probability of selection for the top 90 spike sites that have undergone diversifying selection, grouped by whether they were under selection at the September 30, 2022 analysis date (top) or no longer under selection (bottom).

SUPPLEMENTARY FIGURE S3. Related to Figure 3.

(A) Boxplot of the number of possible amino acid substitutions that decrease ACE2 binding for each position in the RBD, grouped by whether the site is under diversifying (red), purifying (blue), or neutral (grey) selection.

(B) Deep mutational scan site total escape values from convalescent plasma, antibodies from Moderna vaccinated patients, and therapeutic antibodies for sites under diversifying (red), purifying (blue), or neutral (grey) selection.

(C-D) For each possible amino acid substitution within the RBD, scatterplot of the effect on ACE2 binding, effect on RBD expression, and average escape from convalescent antibodies.

SUPPLEMENTARY FIGURE S4. Related to Figure 4.

(A) Number of isolates from the pre-vaccination era (collected on or before December 31, 2020) with any combinations of RBD mutations which confer increased ACE2 binding (L452*, E484*, K417*, G446*, N501*, S477*, T478K), escape from convalescent antibodies (L452*, E484*, K417*, G446V), loss of ACE2 binding (loss) (K417*), or neither (no change) (A520S, A522*).

(B) For each US state, correlation of the CDC seroprevalence estimates from January 24, 2021 with the incident rate (cases per 100,000) corresponding to three weeks prior (December 26, 2020).

SUPPLEMENTARY FIGURE S5 Related to Figure 4.

(A-B) Percent of epitopes with high frequency variants (HFM, found in 1000+ isolates), broken down by (A) gene and (B) ORF1ab protein product.

(C-D) Distribution of the number of HFM observed in (A) putative memory CD8+ T cell epitope sequences, and (B) epitopes from RBD and non-RBD regions.

(E-F) Number of putative memory CD8+ T cell epitope sequences that contain sites under (E) diversifying and (F) purifying selection per gene

(G) Distribution of the number of sites under selection in epitopes, broken down by diversifying and negative selection in spike peptides

(top panel) putative CD4 T cell epitopes from SARS-CoV-2 naïve individuals

(middle panel) putative CD8 T cell epitopes from SARS-CoV-2 naïve individuals

(bottom panel) putative CD8 T cell epitopes recovered COVID patients

SUPPLEMENTARY FIGURE S6. Related to Figure 4.

Distribution of the number of sites under purifying and diversifying selection in spike, N, ORFa1, ORF1b, ORF3a, and ORF7a peptides and genome-wide 15-mers. Significance was assessed using a one-sided Wilcoxon rank-sum test.

SUPPLEMENTARY FIGURE S7. Related to Figure 2.

(A) Pearson correlation coefficients of the log probabilities of purifying (upper right) and diversifying (lower left) selection across six different down-sampled replicates.

(B) Scatterplot of the log probabilities of purifying selection between two down-sampled replicates.
(D) Scatterplot of the log probabilities of diversifying selection between two down-sampled replicates.

SUPPLEMENTARY TABLE LEGENDS

SUPPLEMENTARY TABLE S1. (A) Gene-level and (B) protein-level summaries of the HFM and sites under selection.
SUPPLEMENTARY TABLE S2. Comprehensive list of sites under (A) diversifying and (B) purifying selection.
SUPPLEMENTARY TABLE S3. Comprehensive list of sites under diversifying and purifying selection over time.
SUPPLEMENTARY TABLE S4A. GISAID accession IDs for down-sampled set of two million sequences.
SUPPLEMENTARY TABLE S4B. GISAID accession IDs for down-sampled subsets of sequences used for selection analyses and plotting.

Supplementary Figure S1

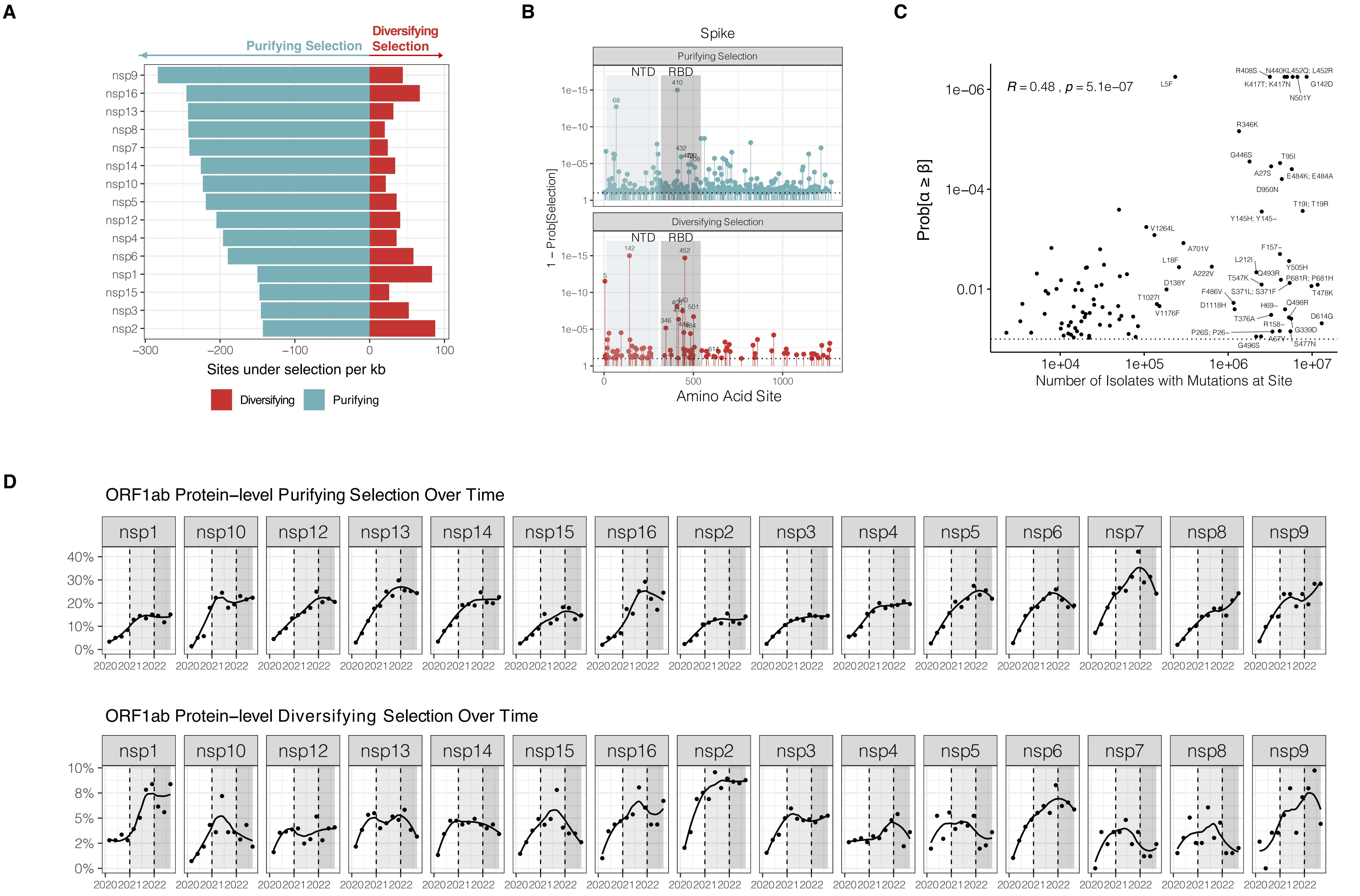

Supplementary Figure S2

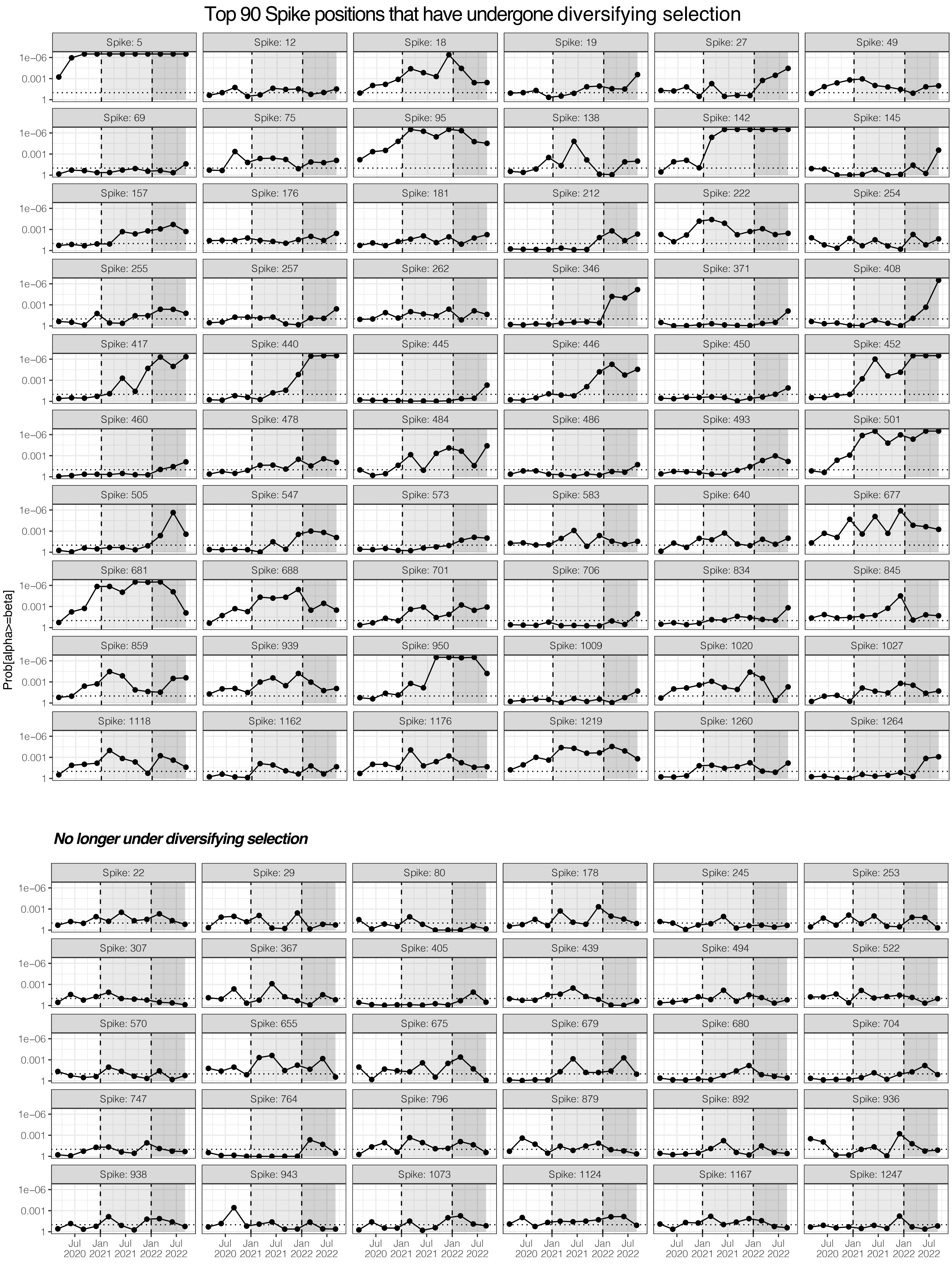

Supplementary Figure S3

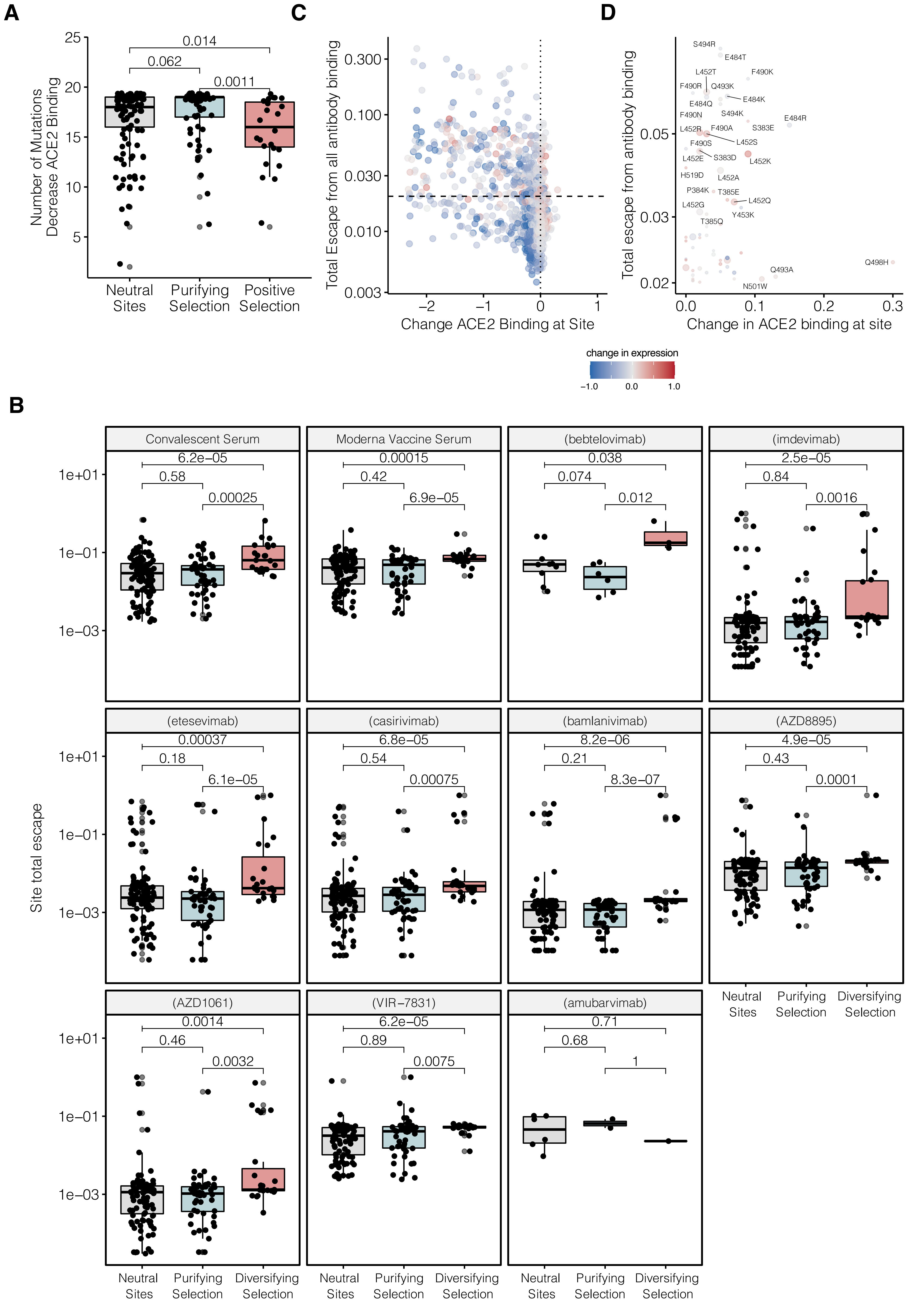

Supplementary Figure S4

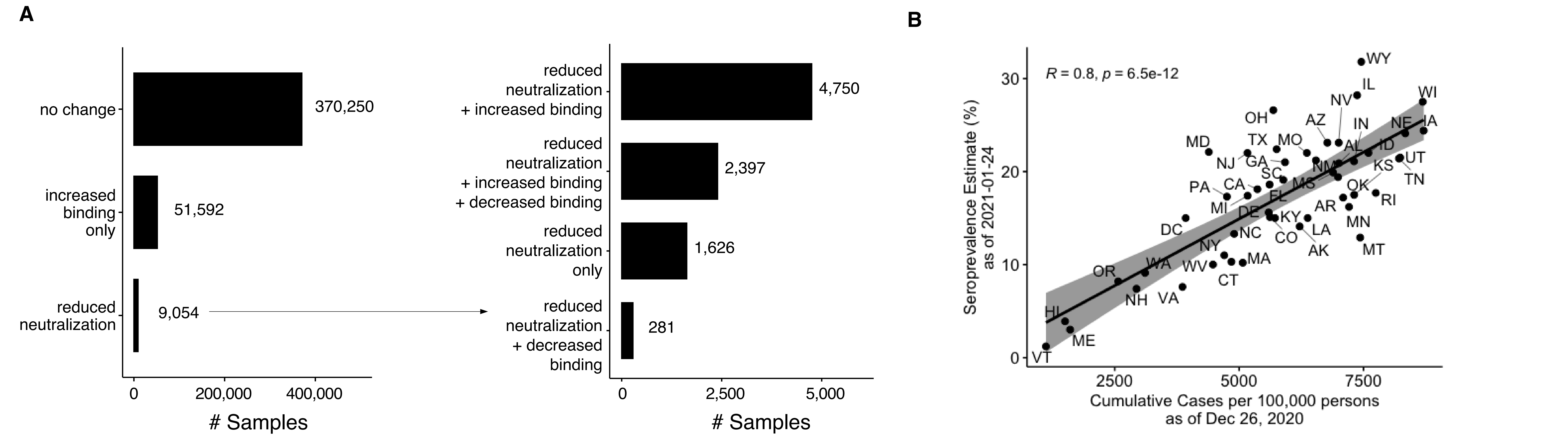

Supplementary Figure S5

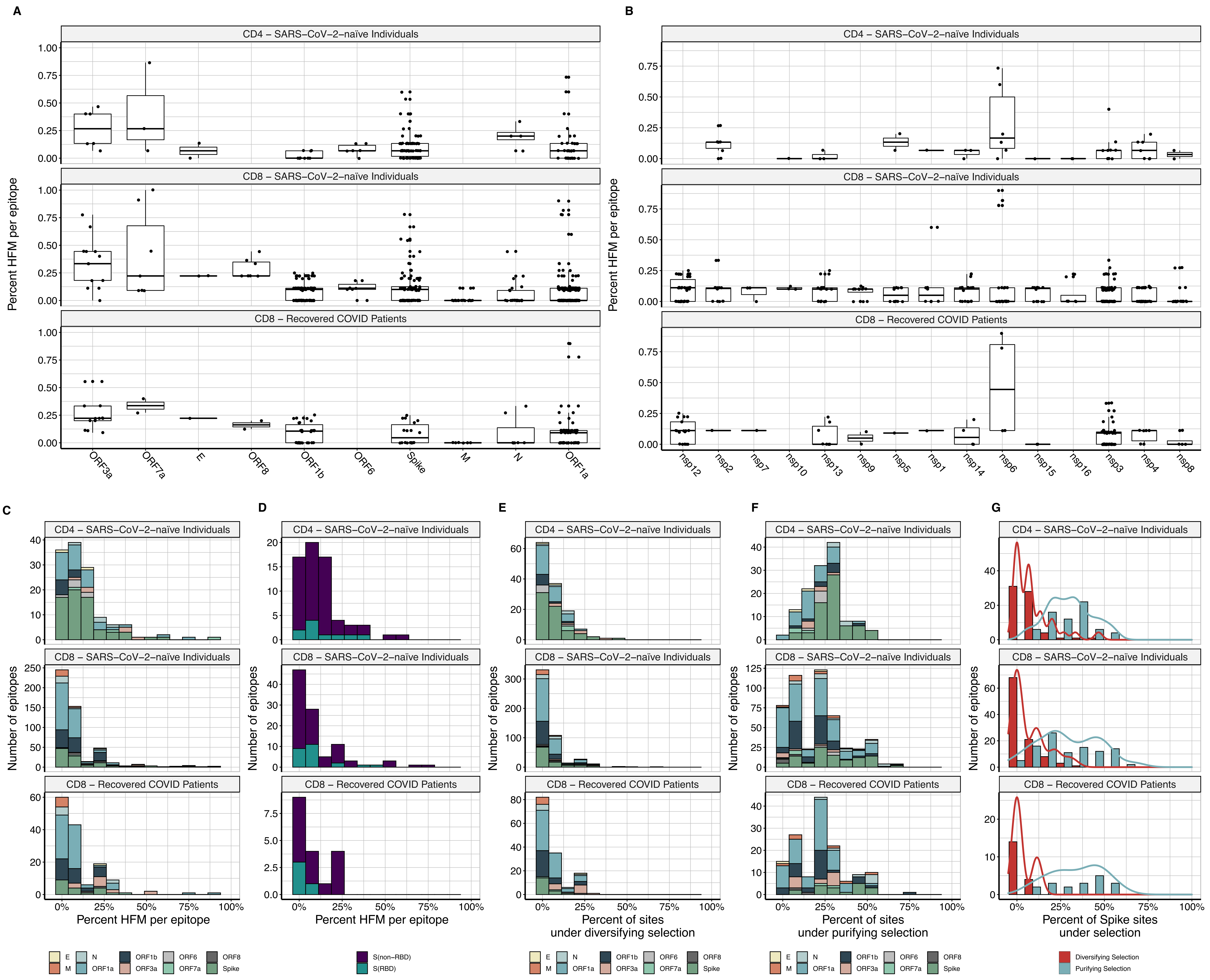

Supplementary Figure S6

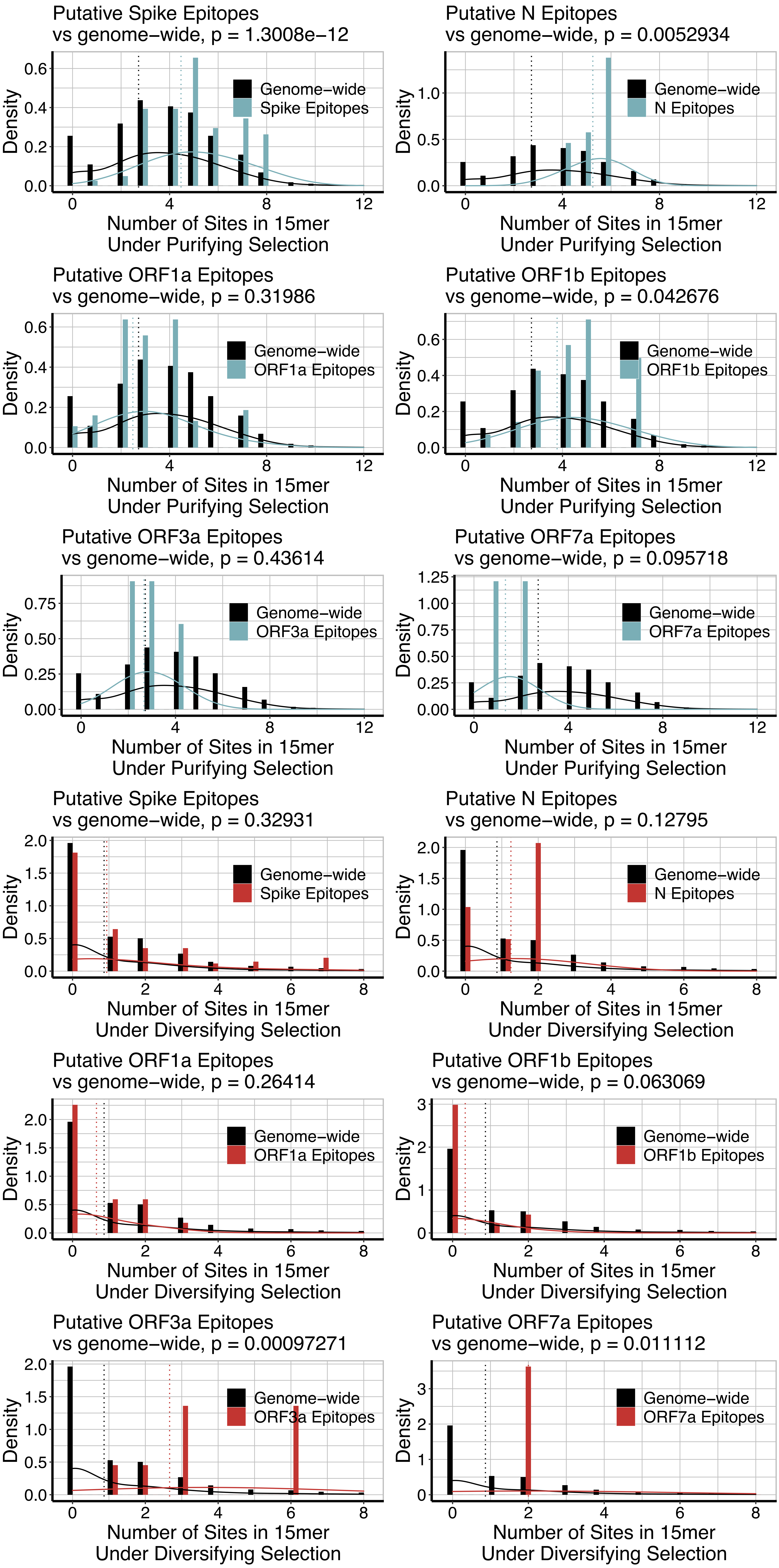

Supplementary Figure S7

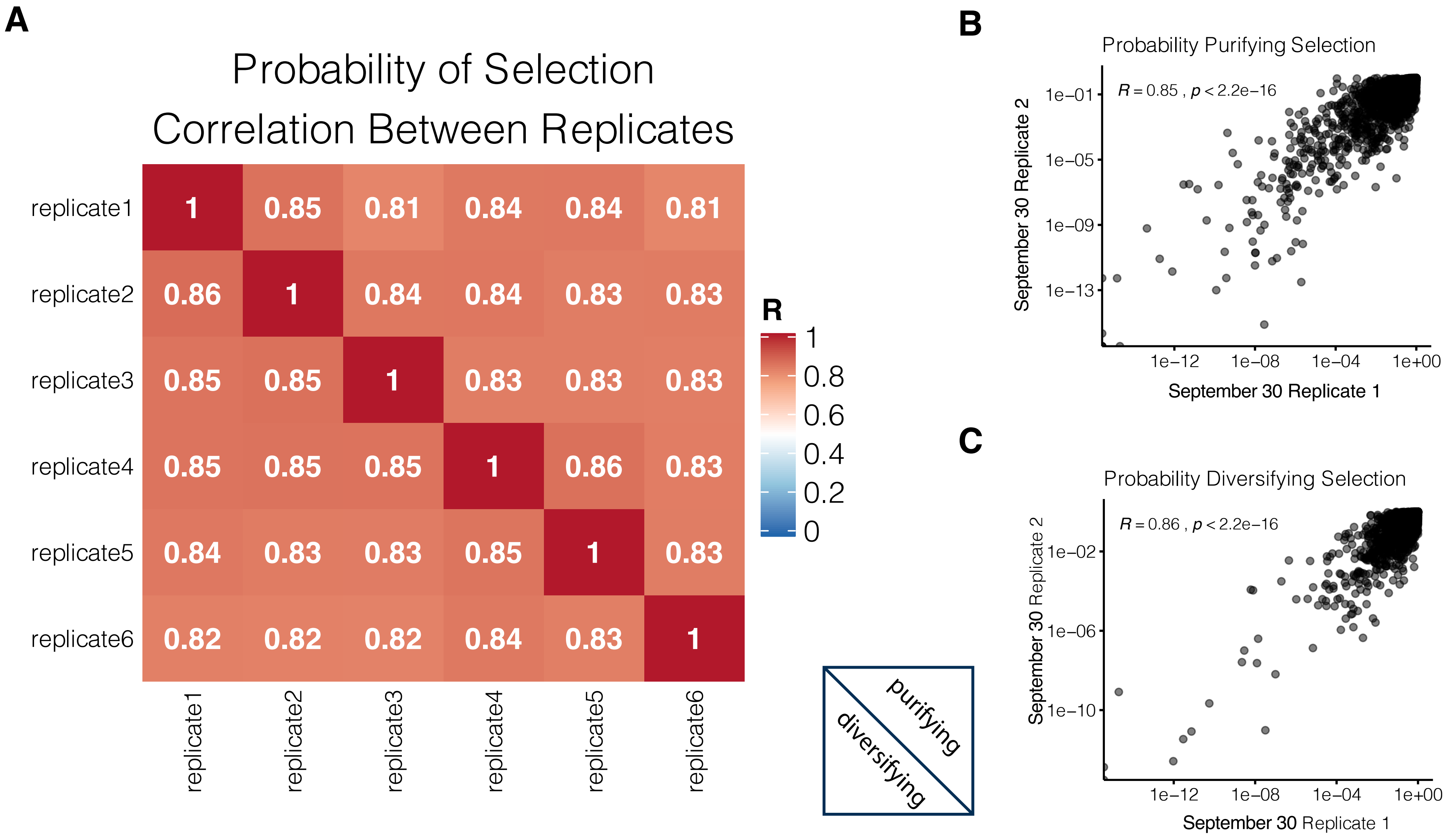

Supplement: Supplementary file 1 — Supplementary Materials [file 44298_2023_7_MOESM1_ESM.pdf]
