# Supplementary material for: Evolutionary trajectory of SARS-CoV-2 genome shifts during widespread vaccination and emergence of Omicron variant
Source: Npj Viruses. 2023 Nov 14;1:5. doi: 10.1038/s44298-023-00007-z (PMC11721106; doi:10.1038/s44298-023-00007-z)
Supplement: Supplementary file 2 — Reporting Summary [file 44298_2023_7_MOESM2_ESM.pdf]

nature portfolio

Corresponding author(s): Copin c3bcd699-4426-4cc2-b662-82351e2268e4

Last updated by author(s): Aug 25, 2023

Reporting Summary

Nature Portfolio wishes to improve the reproducibility of the work that we publish. This form provides structure for consistency and transparency in reporting. For further information on Nature Portfolio policies, see our Editorial Policies and the Editorial Policy Checklist.

Statistics

For all statistical analyses, confirm that the following items are present in the figure legend, table legend, main text, or Methods section.

n/a	Confirmed	
☐	☒	The exact sample size (*n*) for each experimental group/condition, given as a discrete number and unit of measurement
☒	☐	A statement on whether measurements were taken from distinct samples or whether the same sample was measured repeatedly
☐	☒	The statistical test(s) used AND whether they are one- or two-sided<br>*Only common tests should be described solely by name; describe more complex techniques in the Methods section.*
☒	☐	A description of all covariates tested
☒	☐	A description of any assumptions or corrections, such as tests of normality and adjustment for multiple comparisons
☒	☐	A full description of the statistical parameters including central tendency (e.g. means) or other basic estimates (e.g. regression coefficient) AND variation (e.g. standard deviation) or associated estimates of uncertainty (e.g. confidence intervals)
☐	☒	For null hypothesis testing, the test statistic (e.g. *F*, *t*, *r*) with confidence intervals, effect sizes, degrees of freedom and *P* value noted<br>*Give P values as exact values whenever suitable.*
☒	☐	For Bayesian analysis, information on the choice of priors and Markov chain Monte Carlo settings
☒	☐	For hierarchical and complex designs, identification of the appropriate level for tests and full reporting of outcomes
☐	☒	Estimates of effect sizes (e.g. Cohen's *d*, Pearson's *r*), indicating how they were calculated

Our web collection on statistics for biologists contains articles on many of the points above.

Software and code

Policy information about availability of computer code

Data collection	Sequences were downloaded from the GISAID EpiCoV database.<br>Data from deep mutational scans was downloaded from https://github.com/jbloomlab/SARS-CoV-2-RBD_DMS_Omicron (RBD binding, expression constraints) and https://github.com/jbloomlab/SARS2_RBD_Ab_escape_maps (escape maps).<br>CDC seroprevalence estimates were downloaded from https://covid.cdc.gov/covid-data-tracker/#serology-surveillance.<br>The John Hopkins daily incident rate estimates were downloaded from https://github.com/CSSEGISandData/COVID-19.<br>Previously published T cell epitope sequences were downloaded from Supplementary Table 1 of Mateus et al., 2020 and supplementary Tables 4 and 7 of Saini et al., 2021.
Data analysis	The selection analysis of of sequences was performed using FUBAR (Analysis Version: 2.2) within the HyPhy (2.5.8(MPI)) package.<br>Custom code was generated to downsample sequences to input into these analyses and generate the analysis of the selection analysis results, which will be made available in the following repository: https://github.com/regeneron-mpds/SARS-CoV-2_evolution.

For manuscripts utilizing custom algorithms or software that are central to the research but not yet described in published literature, software must be made available to editors and reviewers. We strongly encourage code deposition in a community repository (e.g. GitHub). See the Nature Portfolio guidelines for submitting code & software for further information.

Data

Policy information about availability of data

All manuscripts must include a data availability statement. This statement should provide the following information, where applicable:

- Accession codes, unique identifiers, or web links for publicly available datasets
- A description of any restrictions on data availability
- For clinical datasets or third party data, please ensure that the statement adheres to our policy

All data used in this paper was from publicly available sources. Links to those sources and associated accession IDs are provided in the text and supplemental tables.

Research involving human participants, their data, or biological material

Policy information about studies with human participants or human data. See also policy information about sex, gender (identity/presentation), and sexual orientation and race, ethnicity and racism.

Reporting on sex and gender	We do not report on sex or gender.
Reporting on race, ethnicity, or other socially relevant groupings	We do not report on race or ethnicity.
Population characteristics	Our approach incorporates protections for lineages, which are linked to geographic locations.
Recruitment	Our study utilizes publicly available SARS-CoV-2 sequences from GISAID.
Ethics oversight	Our study utilizes publicly available SARS-CoV-2 sequences from GISAID.

Note that full information on the approval of the study protocol must also be provided in the manuscript.

Field-specific reporting

Please select the one below that is the best fit for your research. If you are not sure, read the appropriate sections before making your selection.

☐ Life sciences ☐ Behavioural & social sciences ☒ Ecological, evolutionary & environmental sciences

For a reference copy of the document with all sections, see nature.com/documents/nr-reporting-summary-flat.pdf

Ecological, evolutionary & environmental sciences study design

All studies must disclose on these points even when the disclosure is negative.

Study description	We conducted a comprehensive population genetic study of over thirteen million SARS-CoV-2 genome sequences, collected over a timeframe of approximately three years.
Research sample	We analyzed all full-length sequences available in GISAID that had accompanying location and collection data metadata available at the time of our study.
Sampling strategy	A novel down-sampling strategy was developed to enable selection analyses to be performed, which was not feasible with 13 million sequences. This down sampling procedure was performed in a way that preserved lineages and collection dates, to try to ensure that a representative dataset was generated.
Data collection	Previously generated, publicly available data was utilized for this study
Timing and spatial scale	Sequences from 2020-2022 were utilized in this analysis. The down-sampling procedure that was developed ensured that representatives from different collection periods were preserved. Additionally, a historical analysis was performed to analyze how evolutionary forces have shifted over time.
Data exclusions	Low quality sequences were excluded.
Reproducibility	To ensure that the down sampling procedure generated representative sets of sequences, this process was repeated and its impact on the analysis was assessed and provided as a supplemental figure.
Randomization	This study is an analysis publicly available sequences, so randomization is not relevant here.
Blinding	This study is an analysis publicly available sequences, so blinding is not relevant here.

Did the study involve field work? ☐ Yes ☒ No

Reporting for specific materials, systems and methods

We require information from authors about some types of materials, experimental systems and methods used in many studies. Here, indicate whether each material, system or method listed is relevant to your study. If you are not sure if a list item applies to your research, read the appropriate section before selecting a response.

Materials & experimental systems

n/a	Involved in the study
☒	☐ Antibodies
☒	☐ Eukaryotic cell lines
☒	☐ Palaeontology and archaeology
☒	☐ Animals and other organisms
☒	☐ Clinical data
☒	☐ Dual use research of concern
☒	☐ Plants

Methods

n/a	Involved in the study
☒	☐ ChIP-seq
☒	☐ Flow cytometry
☒	☐ MRI-based neuroimaging
